# Supplementary material for: Associations of Neonatal Dairy Calf Faecal Microbiota with Inflammatory Markers and Future Performance
Source: Animals (Basel). 2024 Aug 31;14(17):2533. doi: 10.3390/ani14172533 (PMC11394540; doi:10.3390/ani14172533)
Supplement: Supplementary file 1 [file animals-14-02533-s001.zip › animals-3162593-supplementary.pdf]

Associations of Neonatal Dairy Calf Faecal Microbiota with Inflammatory Markers and Future Performance

Marina Loch, Elisabeth Dorbek-Sundström, Aleksi Husso, Tiina Pessa-Morikawa, Tarmo Niine, Tanel Kaart, Kerli Mõtus, Mikael Niku and Toomas Orro

Supplemental Materials

Abbreviations:

W1: week 1 of life, ages 1-7 days

W2: week 2 of life, ages 8-14 days

W3: week 3 of life, ages 15-21 days

HL: halofuginone lactate

Incorrect treatment: started >48h after birth and/or lasted less than 7 days

Correct treatment: according to manufacturer's protocol of one daily oral dose for 7 days starting <48 hours after birth

Hp: haptoglobin

IL-6: interleukin-6

SAA: serum amyloid A

TNF-α: tumour necrosis factor-alpha

Total reads: total 16S sequencing read counts in samples

For all regression models: Variables remained in models even when not significant if they showed confounding effect by changing the coefficient by ≥10% when being removed from the model. All regression models refer to genus-level abundance.

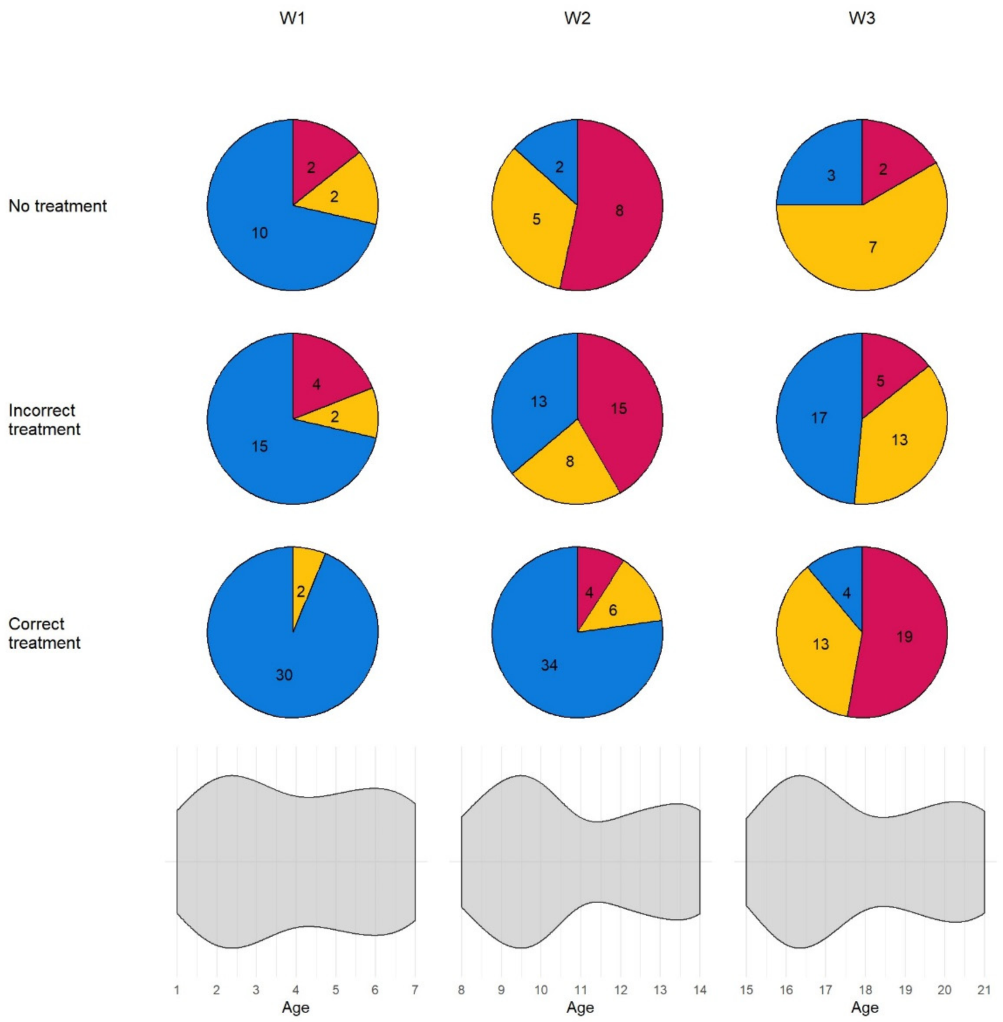

Supplemental Figure S1: Number of calves per week-group and treatment group: blue: no *Cryptosporidium* spp. oocysts, yellow: low *Cryptosporidium* spp. oocyst count, pink: high *Cryptosporidium* spp. oocyst count. Below, the figure shows the age distribution of calves within each week-group.

Supplemental Table S1. Number of calves per group based on *Cryptosporidium* spp. oocyst count and halofuginone lactate (HL) treatment

		W1 (n)	W2 (n)	W3 (n)
Cryptosporidium spp. opg	No oocysts	55	49	24
	Low opg	6	19	33
	High opg	6	27	26

HL treatment group	No treatment	14	15	12
	Incorrect treatment	21	36	35
	Correct treatment	32	44	36
Total number of calves per week-group		67	95	83

Opg, oocysts per gram of faeces; low opg, below median of the week of age; high opg, above median of the week of age; HL, halofuginone lactate; incorrect treatment, started >48h after birth and/or lasted <7 days; correct treatment, according to manufacturer's instructions of one daily dose of HL for7 days starting <48 h after birth

Supplemental materials regarding W1 (group of one-week old calves, ages 1-7 days)

Supplemental Table S2. Association of *Erysipelatoclostridium* abundance with SAA concentration category in W1 (n = 67). Results of negative binomial regression. Dispersion = mean; LR chi2(8) = 33.49; Prob > chi2 = 0.0001; Log likelihood = -237.12235; Pseudo R2 = 0.0660; LR test of alpha = 0: chibar2(01) = 9657.24; Prob >= chibar2 <0.001.

		n	Coef.	SEM	p-value	95% CI
SAA concentration category	low	22	0			
	moderate	22	2.38	0.77	0.002	0.87; 3.90
	high	23	2.97	0.69	<0.001	1.63; 4.32
Age in days	1	7	0			
	2	13	4.73	1.31	<0.001	2.15; 7.30
	3	11	4.38	1.36	0.001	1.71; 7.05
	4	7	4.78	1.39	0.001	2.05; 7.50
	5	9	4.39	1.35	0.001	1.74; 7.03
	6	10	2.23	1.35	0.098	-0.41; 4.87
	7	10	5.52	1.39	<0.001	2.80; 8.23
_cons			-13.63	1.25	<0.001	-16.08; -11.18
ln(total reads)			1 (exposure)			
/lnalpha			1.36	0.19		0.99; 1.73
alpha			3.88	0.739		2.68; 5.61

Supplemental Table S3. Association of *Megasphaera* abundance with IL-6 concentration groups in W1 (n = 67). Results of negative binomial regression. Dispersion =mean; LR chi2(3) = 25.39, Prob > chi2 <0.001; Log likelihood = -138.83336; Pseudo R2 = 0.0838; LR test of alpha = 0: chibar2(01) = 9587.94.

		n	Coef.	SEM	p-value	95% CI
IL-6 concentration group	Low	23	0			
	Moderate	22	5.61	1.78	0.002	2.13; 9.10
	High	22	8.44	2.05	<0.001	4.42; 12.47
Age in days (continuous)			3.07	0.70	<0.001	1.69; 4.45
_cons			-27.51	4.48	<0.001	-36.30; -18.72
ln (total reads)			1	(exposure)		
/lnalpha			2.35	0.28		1.81; 2.89

alpha			10.52	2.90		6.12; 18.07

Supplemental Table S4. Association of *Gallibacterium* abundance with IL-6 concentration group in W1 (n = 67). Results of negative binomial regression. Dispersion = mean; Log likelihood = -401.01192; LR chi2(5) = 26.99; Prob > chi2 = 0.0001; Pseudo R2 = 0.0326; LR test of alpha = 0: chibar2(01) = 3.2e+05.

		n	Coef.	SEM	p-value	95% CI
IL-6 concentration group	Low	23				
	Moderate	22	-2.95	0.70	<0.001	-4.33; -1.57
	high	22	-2.33	0.72	0.001	-3.76; -0.90
Age in days (continuous)			0.36	0.20	0.079	-0.04; 0.75
Cryptosporidium spp. oocyst count group	No oocysts	55				
	Below median of W1	6	-1.18	1.02	0.244	-3.17; 0.81
	Above median of W1	6	-0.77	1.01	0.445	-2.75; 1.21
_cons			-4.10	0.98	<0.001	-6.03; -2.17
ln(total reads)			1 (exposure)			
/lnalpha			1.60	0.16		1.29; 1.90

Supplemental Table S5. Association of *Escherichia-Shigella* abundance with IL-6 concentration groups (n = 67). Results of negative binomial regression. LR chi2(8) = 30.84; dispersion = mean; Prob > chi2 = 0.0001; Log likelihood = -665.74021; Pseudo R2 = 0.0226; LR test of alpha = 0: chibar2(01) = 6.8e+05; Prob >= chibar2 <0.001.

		n	Coef.	SEM	p-value	95% CI

IL-6 concentration group	Low-moderate	Low n= 23	-0.22	0.45	0.628	-1.09; 0.66
	Moderate-high	Moderate n = 22	1.31	0.41	0.001	0.50; 2.123
	Low-high	High n = 22	1.10	0.439	0.011	0.25; 1.94
Age in days	1	7	0			
	2	13	-1.39	0.59	0.018	-2.54; -0.24
	3	11	-1.19	0.58	0.039	-2.32; -0.06
	4	7	-2.30	0.69	0.001	-3.65; -0.96
	5	9	-1.92	0.62	0.002	-3.14; -0.70
	6	10	-3.07	0.63	<0.001	-4.32; -1.83
	7	10	-1.45	0.71	0.041	-2.85; -0.06
_cons			-0.50	0.61	0.413	-1.69; 0.69
ln(total reads)			1(exposure)			
/lnalpha			0.328701	0.15		0.04; 0.62
alpha			1.389162	0.21		1.04; 1.86

Supplemental Table S6. Associations of calving-conception interval with logarithmically transformed abundances of *Megasphaera* (present in n = 17 cows) and *Gallibacterium* (present in n = 47 cows) in W1. Results of negative binomial regression, n = 58; LR chi2(4) = 8.36; Dispersion = mean; Prob > chi2 = 0.0791; Log likelihood = -307.45642; Pseudo R2 = 0.0134; LR test of alpha = 0: chibar2(01) = 1534.58; Prob >= chibar2 <0.001.

		n	Coef.	SEM	p-value	95% CI
log(*Megasphaera*)			0.07	0.03	0.028	0.01; 0.14
log(*Gallibacterium*)			-0.07	0.03	0.014	-0.12; -0.01
HL treatment group	No treatment	13				
	Incorrect treatment	20	0.11	0.18	0.549	-0.25; 0.47
	Correct treatment	25	-0.04	0.17	0.836	-0.37; 0.30
_cons			4.80	0.19	<0.001	4.44; 5.17
/lnalpha			-1.44	0.19		-1.80; -1.07
alpha			0.24	0.04		0.17; 0.34

Supplemental materials regarding W2 (group of two-week old calves, ages 8-14 days)

Supplemental Table S7. Association of *Collinsella* abundance with SAA concentration group in W2 (n = 95). Results of negative binomial regression. LR chi2(10) = 44.67; dispersion = mean; Prob > chi2 <0.001; Log likelihood = -648.35447; Pseudo R2 = 0.0333; LR test of alpha = 0: chibar2(01) = 9.5e+04; Prob >= chibar2 <0.001.

		n	Coef.	SEM	p-value	95% CI
SAA concentration group	Low-moderate	Low n = 31	-2.32	0.39	<0.001	-3.08; -1.57
	Moderate-high	Moderate n = 32	1.51	0.38	<0.001	0.78; 2.25
	Low-high	High n = 32	-0.81	0.42	0.052	-1.63; 0.01
Age in days	8	10	0			
	9	20	0.28	0.52	0.589	-0.74; 1.31
	10	22	0.91	0.55	0.098	-0.17; 1.99
	11	4	-0.67	0.80	0.404	-2.234; 0.90
	12	11	0.24	0.58	0.678	-0.89; 1.38
	13	12	-0.77	0.64	0.229	-2.03; 0.48
	14	16	0.06	0.60	0.923	-1.12; 1.23
Cryptosporidium spp. oocyst count	No oocysts	49	0			
	Below median of W2	19	-1.23	0.40	0.002	-2.01; -0.44
	Above median of W2	27	-0.45	0.34	0.187	-1.11; 0.22
_cons			-3.31	0.54	<0.001	-4.37; -2.25
ln(total reads)			1 (exposure)			
/lnalpha			0.53	0.12		0.28; 0.77

Supplemental Table S8. Association of *Collinsella* abundance with Hp concentration groups in W2 (n = 95). Results of negative binomial regression; LR chi2(8) = 28.39; dispersion = mean; Prob > chi2 = 0.0004; Log likelihood = -656.49628; Pseudo R2 = 0.0212; LR test of alpha = 0: chibar2(01) = 1.1e+05; Prob >= chibar2 <0.001.

		n	Coef.	SEM	p-value	95% CI
Hp concentration group	Low-moderate	Low n = 31	1.23	0.43	0.004	0.39; 2.07
	Moderate-high	Moderate n = 32	-2.12	0.42	<0.001	-2.95; -1.29
	Low-high	High n = 32	-0.89	0.36	0.015	-1.60; -0.17
Age in days	8	10				
	9	20	-0.8	0.61	0.186	-1.99; 0.39
	10	22	-0.06	0.54	0.909	-1.11; 0.99
	11	4	-0.003	0.82	0.997	-1.61; 1.60
	12	11	-1.20	0.64	0.063	-2.46; 0.07
	13	12	-1.33	0.61	0.03	-2.53; -0.13
	14	16	-0.68	0.61	0.265	-1.87; 0.51
_cons			-3.91	0.51	<0.001	-4.9; -2.91
ln(total reads)			1(exposure)			
/lnalpha			0.65	0.12		0.41; 0.89
alpha			1.91	0.23		1.51; 2.43

Supplemental Table S9. Association of *Succinivibrio* abundance and SAA concentration groups in W2 (n = 95). Results of negative binomial regression; LR chi2(12) = 23.20; dispersion = mean; Prob > chi2 = 0.0261; Log likelihood = -178.22062; Pseudo R2 = 0.0611; LR test of alpha = 0: chibar2(01) = 4442.07; Prob >= chibar2 <0.001

		n	Coef.	SEM	p-value	95% CI
SAA concentration group	Low-moderate	Low n = 31	4.32	1.48	0.003	1.42; 7.21
	Moderate-high	Moderate n = 32	-6.77	2.08	0.001	-10.85; -2.68
	Low-high	High n = 32	-2.45	2.06	0.235	-6.48; 1.59
Age in days	8	10	0			
	9	20	-4.72	2.25	0.036	-9.13; -0.32
	10	22	-1.54	1.91	0.419	-5.28; 2.20

	11	4	9.02	4.03	0.025	1.13; 16.92
	12	11	-1.48	2.39	0.538	-6.16; 3.22
	13	12	3.81	2.38	0.108	-0.84; 8.47
	14	16	-1.41	1.85	0.445	-5.04; 2.21
Cryptosporidium spp. oocyst count	No oocysts	49				
	Below median of W2	19	1.02	1.45	0.482	-1.82; 3.86
	Above median of W2	27	-2.60	2.05	0.204	-6.61; 1.41
HL treatment group	None	15	0			
	Incorrect	36	5.05	2.4872 12	0.042	0.18; 9.93
	correct	44	3.67	2.3196 52	0.113	-0.87; 8.22
_cons			-12.47	2.58	<0.001	-17.51; -7.42
ln(total reads)			1(exposur e)			
/lnalpha			2.40	0.23		1.95; 2.86
alpha			11.08	2.55		7.05; 17.41

Supplemental Table S10. Association of *Flavonifractor* abundance with Hp concentration groups inW2 (n = 95). Results of negative binomial regression; LR chi2(12) = 35.41; dispersion = mean; Prob > chi2 = 0.0004; Log likelihood = -215.5995; Pseudo R2 = 0.0759; LR test of alpha = 0: chibar2(01) = 2486.20; Prob >= chibar2 <0.001.

		n	Coef.	SEM	p-value	95% CI
Hp concentration group	Low-moderate	Low n = 31	-0.45	0.86	0.603	-2.13; 1.24
	Moderate-high	Moderate n = 32	-2.73	1.0	0.006	-4.69; -0.78
	Low-high	High n = 32	-3.18	0.83	<0.001	-4.81; -1.55
Age in days	8	10	0			
	9	20	0.52	1.46	0.723	-2.35; 3.39
	10	22	1.96	1.26	0.12	-0.51; 4.42
	11	4	1.16	1.98	0.556	-2.71; 5.04

	12	11	3.45	1.50	0.022	0.50; 6.40
	13	12	2.53	1.55	0.102	-0.50; 5.55
	14	16	1.02	1.47	0.486	-1.85; 3.90
Cryptosporidium spp. oocyst count group	No oocysts	49	0			
	Below median of W2	19	-1.14	0.86	0.184	-2.83; 0.5439843
	Above median of W2	27	-2.76	1.30	0.033	-5.30; -0.22
HL treatment group	None	15	0			
	incorrect	36	19.80	856.58	0.982	-1659.08; 1698.67
	correct	44	17.98	856.58	0.983	-1660.9; 1696.85
_cons			-26.69	856.58	0.975	-1705.57; 1652.18
ln(total reads)			1 (exposure)			
/lnalpha			1.77	0.21		1.36; 2.18
alpha			5.89	1.23		3.91; 8.87

Supplemental Table S11. Association of *Peptostreptococcus* abundance with Hp concentration groups in W2 (n = 95). Results of negative binomial regression. LR chi2(12) = 40.73; dispersion = mean; Prob > chi2 = 0.0001; Log likelihood = -330.15316; Pseudo R2 = 0.0581; LR test of alpha = 0: chibar2(01) = 2.9e+04; Prob >= chibar2 <0.001.

		n	Coef.	SEM	p-value	95% CI
Hp concentration groups	low	31	0			
	Moderate	32	3.85	1.19	0.001	1.52; 6.17
	high	32	3.34	0.96	0.001	1.46; 5.23
Age in days	8	10				
	9	20	0.98	1.40	0.482	-1.76; 3.73
	10	22	1.38	1.40	0.324	-1.36; 4.12
	11	4	-0.12	1.67	0.942	-3.40; 3.15
	12	11	-2.53	1.69	0.133	-5.84; 0.77

	13	12	-6.98	1.74	<0.001	-10.38; -3.57
	14	16	-2.81	1.41	0.046	-5.57;-0.05
Cryptosporidium oocyst count group	No oocysts	49				
	Below median of W2	19	-0.49	1.18	0.679	-2.79; 1.82
	Above median of W2	27	1.68	0.96	0.08	-0.20; 3.55
HL treatment group	none	15				
	Incorrect	36	-0.02	1.19	0.986	-2.35; 2.31
	correct	44	1.35	1.09	0.215	-0.78; 3.48
_cons			-9.66	1.31	0	-12.22; -7.10
ln(total count)			1 (exposure)			
/lnalpha			1.91	0.17		1.57; 2.25
alpha			6.76	1.18		4.81; 9.52

Supplemental Table S12. Association of *Streptococcus* abundance with Hp concentration groups in W2 (n = 95). Results of negative binomial regression. LR chi2(12) = 27.44; dispersion = mean; Prob > chi2 = 0.0067; Log likelihood = -372.57644; Pseudo R2 = 0.0355; LR test of alpha = 0: chibar2(01) = 9893.15; Prob >= chibar2 <0.001.

		n	Coef.	SEM	p-value	95% CI
Hp concentration group	Low	31	0			
	Moderate	32	2.19	0.59	<0.001	1.03; 3.34
	High	32	0.97	0.64	0.131	-0.29; 2.23
Age in days	8	10	0			
	9	20	-1.28	1.02	0.208	-3.27; 0.71
	10	22	-1.41	0.93	0.129	-3.24; 0.41
	11	4	-1.97	1.29	0.126	-4.49; 0.55
	12	11	-2.21	1.15	0.055	-4.46; 0.04
	13	12	-3.38	1.11	0.002	-5.56; -1.20
	14	16	-1.65	1.09	0.131	-3.78; 0.49
Cryptosporidium spp. oocyst count group	No oocysts	49				

	Below median of W2	19	0.32	0.69	0.643	-1.04; 1.68
	Above median of W2	27	-0.88	0.69	0.203	-2.23; 0.47
HL treatment group	none	15				
	incorrect	36	1.42	0.91	0.118	-0.36; 3.21
	correct	44	0.10	0.91	0.915	-1.68; 1.88
_cons			-6.90	1.03	<0.001	-8.93; -4.88
ln(total reads)			1 (exposure)			
/lnalpha			1.33	0.15		1.03; 1.63
alpha			3.77	0.58		2.79; 5.10

Supplemental Table S13. Association of [*Eubacterium*] *coprostanoligenes* group abundance with IL-6 concentration groups in W2 (n = 95). Results of negative binomial regression. LR chi2(10) = 31.80; dispersion = mean; Prob > chi2 = 0.0004; Log likelihood = -214.51117; Pseudo R2 = 0.0690; LR test of alpha = 0: chibar2(01) = 8384.78; Prob >= chibar2 <0.001.

		n	Coef.	SEM	p-value	95% CI
IL-6 concentration group	low	32	0			
	Moderate	32	-4.92	1.20	<0.001	-7.27; -2.58
	High	31	-3.28	1.22	0.007	-5.66; -0.90
Age in days	8	10	0			
	9	20	2.40	1.54	0.119	-0.62; 5.43
	10	22	0.28	1.64	0.865	-2.93; 3.49
	11	4	0.64	1.89	0.735	-3.07; 4.34
	12	11	3.56	1.70	0.036	0.23; 6.88
	13	12	4.24	1.63	0.009	1.04; 7.44
	14	16	3.51	1.81	0.053	-0.04; 7.07
HL treatment group	none	15	0			
	Incorrect	36	-0.07	1.55	0.965	-3.10; 2.96
	correct	44	2.00	1.36	0.143	-0.67; 4.67
_cons			-9.12	1.68	<0.001	-12.42; -5.82

ln(total reads)			1 (exposure)			
/lnalpha			2.21967	0.22		1.79; 2.65
alpha			9.20	2.03		5.97; 14.18

Supplemental Table S14. Associations of logarithmically abundances of *Collinsella* and *Peptostreptococcus* in W2 with calving-conception interval in days (n = 84). Results of negative binomial regression. LR chi2(5) = 22.22; dispersion = mean; Prob > chi2 = 0.0005; Log likelihood = -439.70588; Pseudo R2 = 0.0246; LR test of alpha = 0: chibar2(01) = 2044.63 Prob >= chibar2 <0.001.

		n	Coef.	SEM	p-value	95% CI
Log(*Collinsella* abundance)			-0.11	0.03	<0.001	-0.17; -0.05
Log(*Peptostreptococcus* abundance)			0.06	0.02	0.01	0.01; 0.11
HL treatment group	None	15	0			
	incorrect	36	0.06	0.15	0.681	-0.23; 0.36
	correct	44	0.12	0.15	0.433	-0.18; 0.42
Age in days (continuous)			0.08	0.03	0.004	0.03; 0.14
_cons			4.09	0.40	<0.001	3.32; 4.87
/lnalpha			-1.53	0.16		-1.84; -1.23
alpha			0.22	0.03		0.16; 0.29

Supplemental Table S15. Association of *Peptostreptococcus* abundance in W2 with one-year average daily weight gain (g/d; n = 95). Results of linear regression. Adjusted $R^2 = 0.19$

		n	Coef.	SEM	p-value	95% CI
Log(*Peptostreptococcus* abundance)			-10.58	3.30	0.002	-17.13; -4.02
Cryptosporidium spp. oocyst count group	No oocysts	49				
	Below median of W2	19	-31.12	20.93	0.141	-72.72; 10.47

	Above median of W2	27	19.24	20.27	0.345	-21.04; 59.52
Age in days (continuous)			-5.27	4.08	0.200	-13.39; 2.84
Hl treatment group	None	15				
	Incorrect	36	-59.80	22.50	0.009	-104.51; -15.08
	correct	44	-85.47	24.26	0.001	-133.69; -37.25
_cons			920.24	52.30	<0.001	816.31; 1024.18

<u>Supplemental materials regarding W3 (group of three-week old calves, ages 15-21 days)</u>

Supplemental Table S16. Association of *Dorea* abundance with Hp concentration groups in W2 (n = 83). Results of negative binomial regression. LR chi2(8) = 17.35; dispersion = mean; Prob > chi2 = 0.0267; Log likelihood = -436.19908; Pseudo R2 = 0.0195; LR test of alpha=0: chibar2(01) = 3.4e+04; Prob >= chibar2 < 0.001.

		n	Coef.	SEM	p-value	95% CI
Hp concentration group	low	28	0			
	moderate	28	1.76	0.51	0.001	0.76; 2.75
	high	27	1.27	0.58	0.027	0.14; 2.40
Age in days	15	7	0			
	16	20	-0.40	0.91	0.656	-2.18; 1.37
	17	16	-0.52	0.88	0.551	-2.24; 1.20
	18	6	0.14	1.01	0.888	-1.84; 2.13
	19	9	-0.69	0.91	0.446	-2.47; 1.09
	20	12	-0.91	0.98	0.353	-2.82; 1.01
	21	13	-1.55	0.91	0.09	-3.34; 0.24
_cons			-6.15	0.87	<0.001	-7.87; -4.44
ln(total reads)			1 (exposure)			
/lnalpha			1.14	0.14		0.86; 1.42
alpha			3.14	0.45		2.37; 4.15

Supplemental Table S17. Association of *Erysipelotrichaceae* UCG-004 abundance with IL-6 concentration groups in W3 (n = 83). Results of negative binomial regression. LR chi2(10) = 38.93; dispersion = mean; Prob > chi2 <0.0001; Log likelihood = -172.80823; Pseudo R2 = 0.1012; LR test of alpha = 0: chibar2(01) = 7610.37; Prob >= chibar2 <0.001.

		n	Coef.	SEM	p-value	95% CI
IL-6 concentration group	Low-moderate	Low n = 29	0.89	1.29	0.489	-1.63; 3.41
	Moderate-high	Moderate n = 27	-4.47	1.29	0.001	-6.99; -1.94
	Low-high	High n = 27	-3.58	1.19	0.003	-5.92; -1.23
Age in days	15	7				
	16	20	-6.60	1.58	<0.001	-9.69; -3.50
	17	16	-1.37	1.66	0.408	-4.62;1.88
	18	6	-0.12	2.10	0.953	-4.23; 3.98
	19	9	-3.48	1.50	0.02	-6.41; -0.54
	20	12	-0.73	1.46	0.618	-3.59; 2.13
	21	13	-1.61	1.47	0.272	-4.49; 1.26
Cryptosporidium spp. oocyst count group	No oocysts	24				
	Below median of W3	33	2.01	1.05	0.055	-0.04; 4.07
	Above median of W3	26	-2.65	1.39	0.056	-5.37; 0.07
_cons			-5.99	1.31	<0.001	-8.56; -3.41
ln(total reads)			1 (exposure)			
/lnalpha			2.10	0.23		1.65; 2.56
alpha			8.20	1.89		5.22; 12.87

Supplemental Table S18. Association of *Erysipelotrichaceae* UCG-004 abundance with TNF-α concentration groups in W3 (n = 83). Results of negative binomial regression. LR chi2(12) = 50.92; dispersion = mean; Prob > chi2 < 0.0001; Log likelihood = -166.81548; Pseudo R2 = 0.1324; LR test of alpha = 0: chibar2(01) = 4743.28; Prob >= chibar2 <0.001.

		n	Coef.	SEM	p-value	95% CI
TNF-α concentration group	Low-moderate	Low n = 29	0.02	1.30	0.988	-2.52; 2.56

	Moderate -high	Moderate n = 26	-6.95	2.09	0.001	-11.05; -2.85
	Low-high	High n = 28	-6.93173	1.70	<0.001	-10.27; -3.59
Age in days	15	7	0			
	16	20	-7.12	1.88	<0.001	-10.80; -3.43
	17	16	-2.86	2.26	0.206	-7.29; 1.57
	18	6	-0.01	2.87	0.997	-5.64; 5.62
	19	9	-5.19	1.74	0.003	-8.61; -1.78
	20	12	-4.03	2.31	0.081	-8.56; 0.50
	21	13	-4.45	2.22	0.045	-8.80; -0.10
Cryptosporidium spp. oocyst count group	No oocysts	24	0			
	Below median of W3	33	-0.40	1.63	0.805	-3.60; 2.80
	Above median of W3	26	-3.87	1.26	0.002	-6.34; -1.40
HL treatment group	none	12	0			
	Incorrect	35	0.19	1.66	0.910	-3.06; 3.44
	correct	36	1.17	1.53	0.444	-1.83; 4.17
_cons			-3.37	2.63	0.200	-8.52; 1.78
ln(total reads)			1 (exposure)			
/lnalpha			1.85	0.23		1.39; 2.31
alpha			6.36	1.49		4.02; 10.05

Supplemental Table S19. Association of *Bilophila* abundance with TNF-α concentration groups in W3 (n = 83). Results of negative binomial regression; LR chi2(12) = 18.63; dispersion = mean; Prob > chi2 = 0.0978; log likelihood = -214.51982; Pseudo R2 = 0.0416; LR test of alpha = 0: chibar2(01) = 2668.01; Prob >= chibar2 <0.001

		n	Coef.	SEM	p-value	95% CI
TNF-α concentration group	Low-moderate	Low n= 29	-0.37	0.89	0.680	-2.10; 1.37

	moderate -high	Moderate n = 26	-2.88	0.84	0.001	-4.53; -1.23
	Low-high	High n = 28	-3.25	0.92	<0.001	-5.05; -1.44
Age in days	15	7	0			
	16	20	2.62	1.47	0.076	-0.27; 5.51
	17	16	2.45	1.38	0.076	-0.25; 5.145
	18	6	-0.22	1.79	0.904	-3.73; 3.30
	19	9	1.59	1.42	0.260	-1.18; 4.37
	20	12	1.86	1.31	0.156	-0.71; 4.43
	21	13	0.18	1.56	0.906	-2.88; 3.24
Cryptosporidium spp. oocyst count group	No oocysts	24				
	Below median of W3	33	1.63	0.92	0.076	-0.17; 3.43
	Above median of W3	26	0.01	1.30	0.996	-2.54; 2.56
HL treatment group	None	12				
	Incorrect	35	1.46	0.94	0.12	-0.38; 3.29
	correct	36	1.38	1.10	0.209	-0.77; 3.53
_cons			-10.72	1.58	0	-13.82; -7.62
ln(total reads)			1 (exposure)			
/lnalpha			1.80	0.21		1.40; 2.20
alpha			6.06	1.25		4.05; 9.07

Supplemental Table S20. Associations of logarithmically transformed abundances of *Bilophila* (present in n = 33 calves) and *Dorea* (present in n = 64 calves) in W3 with age at first calving in days (n = 79). Results of linear regression. Adjusted R2 = 0.1341

		n	Coef.	SEM	p-value	95% CI
log(*Bilophila* abundance)			-5.32	2.67	0.050	-10.64; -0.01
log(*Dorea* abundance)			-5.47913	2.32	0.021	-10.11; -0.85

HL treatment group	None	12	0			
	Incorrect	35	27.01	12.50	0.034	2.09; 51.94
	correct	32	32.31	13.27	0.017	5.86; 58.76
Age in days (continuous)			1.62	2.11	0.446	-2.59; 5.82
Cryptosporidium spp. oocyst count group	No oocysts	23	0			
	Below median of W3	32	3.96	10.55	0.708	-17.08; 25.01
	Above median of W3	24	11.01	12.71	0.389	-14.33; 36.35
_cons			661.38	42.40	<0.001	576.83; 745.94
